# Deep Learning-Driven Bus Short-Term OD Demand Prediction via a Physics-Guided Adaptive Graph Spatio-Temporal Attention Network

**DOI:** 10.3390/s25216739

**Published:** 2025-11-04

**Authors:** Zhichao Cao, Longfei Song, Silin Zhang, Jingxuan Sun

**Affiliations:** 1 School of Transportation and Civil Engineering, Nantong University, Nantong 226019, China; caozhichao@bjtu.edu.cn (Z.C.); 2133110108@stmail.ntu.edu.cn (L.S.); noct1s@stmail.ntu.edu.cn (J.S.); 2 Engineering Research Center of Sustainable Urban Intelligent Transportation, Ministry of Education, Chengdu 610041, China

**Keywords:** short-term OD demand prediction, small-scale bus dataset, deep learning, PAG-STAN

## Abstract

This study develops a recent model proposed by Zhang et al. to predict bus short-term origin-destination (OD) demand based on a small-scale dataset (i.e., one week’s data per 30 mins’ collecting interval). We distinctively use sole input sequence by introducing a multi-head attention mechanism while simultaneously ensuring prediction accuracy. Extensive experiments demonstrate that one-layer bidirectional LSTMs (BiLSTMs) perform better than multi-layer ones. A modified deep learning model integrating physics-guided mechanisms, adaptive graph convolution, attention networks, and spatiotemporal encoder–decoder is constructed. We retained the original name, i.e., physics-guided adaptive graph spatio-temporal attention network (PAG-STAN) model. The model uses an encoder–decoder architecture, where the encoder captures spatiotemporal correlations via an adaptive graph convolutional LSTM (AGC-LSTM), enhanced by an attention mechanism that adjusts the importance of different spatiotemporal features. The decoder utilizes bidirectional LSTM to reconstruct the periodic patterns and predict the full OD matrix for the next interval. A masked physics-guided loss function, which embeds the quantitative relationship between boarding passenger volume and OD demand, is adopted for training. The Adam optimizer and early stopping technique are used to enhance training efficiency and avoid overfitting. Experimental results show that PAG-STAN outperforms other deep learning models in prediction accuracy. Compared with the suboptimal model, the proposed model achieved reductions of 6.19% in RMSE, 6.59% in MAE, and 8.20% in WMAPE, alongside a 1.13% improvement in R^2^.

## 1. Introduction

The time-dependent demand and passenger flow fluctuations have been investigated for decades. A fluctuation phenomenon is especially pronounced in the context of bus mode. This raises notable tidal/time-varying effects on bus service operations and management. Specifically, a large number of users are willing to take buses during peak hours, while few passengers ride buses during off-peak hours [1]. Such tidal effects originate from commuting activities and are universally observed in urban public transportation systems across the globe. While the intensity may vary with city size, this pattern is equally applicable to Nantong, a typical large city. In practice, a huge number of bus companies have been bearing substantial financial losses and receive government subsidies. As a consequence, accurately learning the OD demand and precisely predicting it are of great practical significance for decision makers [2], not only facilitating better schedules but also benefiting rational planning.

The spatiotemporal distribution of OD demand exhibits complex nonlinear characteristics. However, traditional statistical models such as ARIMA and Kalman filters, while simple in structure and easy to interpret, are limited in handling nonlinear problems due to their assumptions regarding data stationarity and linear relationships [3]. Traditional machine learning models, such as random forests and support vector machines, are highly sensitive to data quality, easily affected by outliers, and fail to fully capture the deep spatiotemporal dependencies inherent in OD matrices [4].

Recent AI learning-related techniques have been advanced and applied for OD demand prediction. Nonetheless, bus OD demand prediction studies have been limited to date, based on a review of the existing literature. Learning the three abilities/merits of handling real-time availability, sparsity, and high-dimensionality issues of the PAG-STAN framework proposed by Zhang et al. [5], we decide to employ this approach to validate its applicability and tractability for predicting bus OD demands instead of metro ones. New modifications including the multi-head attention mechanism and one-layer bidirectional LSTMs are developed to improve generalization on bus OD demand prediction. Validation results show that a small-scale data set is capable of achieving a high-quality short-term prediction accuracy.

### 1.1. Literature Review

The development of short-term traffic/passenger demand prediction can be roughly divided into three categories: statistical-based methods, traditional machine learning-based methods, and deep learning-based methods. Section 1.1 discusses the research features and progress of these three directions.

#### 1.1.1. Statistical-Based Prediction Methods

In prior studies, raw traffic data were typically preprocessed into sequences or vectorized formats, which were subsequently used as inputs for statistical prediction models. Smith and Demetsky [6] used a historical average model to predict future traffic flow in highway traffic prediction problems. Lee and Fambro [7] studied the application of an autoregressive integrated moving average (ARIMA) model in short-term traffic flow prediction. Tan et al. [8] proposed a data aggregation method combining ARIMA and exponential smoothing for short-term traffic flow prediction. Williams and Hoel [9] established a theoretical foundation for modeling univariate traffic condition data streams using seasonal ARIMA and conducted empirical analysis to validate the theoretical assumptions. Emami et al. [10] proposed a Kalman filter-based algorithm that uses connected vehicle data to predict short-term traffic flow at urban intersections in real time, and validated its effectiveness under different connected vehicle penetration rates. Pan et al. [11] proposed an interpretable hybrid model integrating a fundamental diagram and a Markov model to achieve high-precision prediction of traffic flow. However, these statistical models impose strict requirements on time series stability. Given the limitations of focusing on single features in historical data, they struggle to handle the nonlinear and complex traffic patterns, resulting in low-precision short-term demand prediction performance.

#### 1.1.2. Traditional Machine Learning-Based Prediction Methods

With the machine learning approach emerging, transportation demand prediction is no longer confined to the extraction of a single feature. Many studies have developed a variety of machine learning-based models or hybrid approaches to capture and learn the complex features influencing demand. Zhao et al. [12] used a gradient boosting decision tree-based model to analyze the relationship between feature variables and prediction results. Wang et al. [13] divided the 24 h period into four stages based on the bimodal distribution of traffic flow and constructed a prediction model for each stage using random forest. Experimental results showed a good performance as per random forest. Kang et al. [14] developed a hybrid model based on extreme gradient boosting to extract and predict spatiotemporal features of urban travel time. Hong [15] proposed a hybrid prediction model that combines seasonal support vector regression with a chaotic simulated annealing algorithm to handle nonlinear and seasonal fluctuations in traffic flow data. Cai et al. [16] proposed an improved k-nearest neighbors prediction model based on spatiotemporal correlation by introducing equivalent distance and a spatiotemporal state matrix to improve the accuracy of multi-step short-term traffic flow prediction. Chen et al. [17] proposed a short-term traffic flow prediction framework that integrates denoising schemes with artificial neural networks (ANNs). The framework first applies the wavelet filtering, moving average, and Butterworth filtering to denoise loop detector data and then uses ANNs to achieve multi-time scale traffic flow prediction. Raskar and Nema [18] introduced an enhanced hidden Markov model integrated with a metaheuristic optimization algorithm. The model significantly improved the robustness and accuracy of traffic flow prediction under the impact of unexpected events. Jin et al. [19] used a backpropagation neural network to predict metro passenger flow based on an analysis of spatiotemporal complexity features. The predicted results matched well with actual data, demonstrating the effectiveness of the BP neural network. Although these models outperform statistical models in terms of accuracy, there remains the challenge of capturing the complex spatiotemporal dependencies in traffic demand data.

#### 1.1.3. Deep Learning-Based Prediction Methods

Nowadays, compared to traditional machine learning methods, deep learning approaches are more effective at capturing the complex spatiotemporal correlations in big data. Hybrid deep learning methods have become highly advantageous and represent a key research trend in short-term transportation demand prediction. Zhang et al. [20] applied a combined deep learning model using graph convolutional network (GCN) and three-dimensional convolutional neural network (3D CNN) for short-term railway passenger flow prediction. Xia et al. [21] constructed a dynamic spatiotemporal graph convolutional recurrent neural network, which integrates dynamic graph generation and dual-channel convolution mechanisms, achieving significant improvements in traffic flow prediction accuracy across multiple datasets. Zhang et al. [22] proposed a Conv-GCN model that combines multi-graph convolutional network and 3D CNN to capture various periodic features of metro passenger flow, enabling efficient short-term prediction. Shanthappa et al. [23] developed a GCN-based model for bus OD demand prediction. By incorporating land use data around stops to enhance spatial correlation modeling, the model significantly improved prediction performance and stability. Lu et al. [24] employed a Conv-LSTM neural network to predict short-term ride-hailing demand, converting spatiotemporal order data into an image processing problem to effectively capture spatiotemporal features and improve prediction accuracy. Zhang et al. [25] proposed a spatiotemporal residual graph attention network, which incorporates residual graph attention mechanisms and a sequence-to-sequence structure to effectively capture spatiotemporal and periodic features in traffic flow, thereby improving prediction accuracy. He et al. [26] designed a multi-graph convolutional recurrent neural network that integrates multiple graph structures to model heterogeneous correlations between metro stations, achieving high-precision short-term prediction of metro inflow and outflow. Zhan et al. [27] constructed a parallel architecture that combines multi-graph convolutional network and Gated Recurrent Unit (GRU) to model complex spatial relationships between metro stations from the perspectives of adjacency, similarity, and correlation. Yang et al. [28] proposed a multi-task learning model called Res-transformer that integrates transformer and residual networks to jointly predict short-term inflow across multiple modes of transportation, validating the effectiveness of multi-modal joint modeling. Lv et al. [29] proposed an OD passenger flow prediction model based on CNNs and a passenger source attention mechanism, which more accurately identifies and predicts key sources of metro overcrowding, enhancing the targeting and practicality of the predictions. Chen et al. [30] developed a bidirectional spatiotemporal adaptive transformer, which combines dynamic calculation and memory mechanisms to efficiently model and accurately predict complex spatiotemporal dependencies in urban traffic flow. Chu et al. [31] built a novel deep learning model called multi-scale Conv-LSTM, which fully considers temporal and spatial correlations to predict future travel demand and OD flow. Zhao et al. [32] combined GCN and GRU to propose a temporal graph convolutional network model for traffic prediction in urban road networks. Zheng et al. [33] proposed an attention-based Conv-LSTM model that extracts spatial and short-term temporal features by combining CNN and LSTM, and further uses the BiLSTM model to capture daily and weekly periodic features. Noursalehi et al. [34] introduced a novel multi-resolution spatiotemporal neural network to capture spatial and temporal dependencies. Using discrete wavelet transform to decompose demand into different time and frequency components. To address the high dimensionality and sparsity of OD demand data, Zhang et al. [35] proposed a channel-wise attentive split-convolutional neural network, which includes split CNN, channel-wise attention, and inflow/outflow-gated mechanism. Ke et al. [36] introduced a spatiotemporal encoder–decoder residual multi-graph convolutional network to predict ride demand between different OD pairs. Huang et al. [37] proposed a deep learning model based on multi-head spatial attention and bidirectional attention mechanisms for short-term ride-hailing demand prediction.

Reviewing the above abundant literature, indeed relatively few studies focus on bus OD demand prediction. To improve and validate the PAG-STAN model for short-term OD demand prediction on a single bus route, we utilize a small data set to validate its performance and purpose to find out some interesting insights. To the best of our knowledge, our study is a new work to investigate a deep learning model applied to a real-world bus system. Table 1 shows the characteristics of existing literature by comparing studies relevant to our work.

### 1.2. Contributions

In order to further enrich the theoretical framework of deep learning in the field of bus demand prediction. This paper pioneers the application of the PAG-STAN model to short-term OD demand prediction for single bus routes based on a small dataset. To adapt the PAG-STAN model to bus OD prediction, this study has made three key improvements to the original PAG-STAN model.

(i) Unlike the original model, which uses daily, weekly, and real-time input sequences, this study only adopts daily OD data as the input sequence. To fully extract information under a single time scale, the input layer converts daily data into a continuous sequence through temporal concatenation. This design enables the model to focus on the daily periodic features with only daily data dependency, making it more suitable for practical application scenarios with small-scale bus data.
(ii) Unlike the original model, to better encode the spatiotemporal features in daily demand data, this study embeds a multi-head attention module in the encoder. As a result, the model can process the features extracted by the AGC-LSTM layer in parallel within multiple subspaces.
(iii) Experiments show that the performance of using one-layer bidirectional LSTMs in the decoder is better than that of the multi-layer structure, which reduces the risk of overfitting in model training with a small bus dataset.

These innovations extend the applicability of PAG-STAN from metro OD passenger flow prediction to bus demand prediction based on a small dataset.

### 1.3. Outline

The layout of the subsequent sections in this paper is as follows. Section 2 details the architecture and key modules of the PAG-STAN model, including the design and implementation of AGC-LSTM, attention mechanism, and BiLSTM. Section 3 introduces the description of dataset, experimental results, and the comparison with state-of-the-art methods. It evaluates the prediction performance of PAG-STAN against other baseline models and visualizes the model’s prediction effects using charts. Section 4 draws the conclusions and outlines directions for future improvements.

## 2. Methodology

### 2.1. Model Architecture

**Figure 1:**
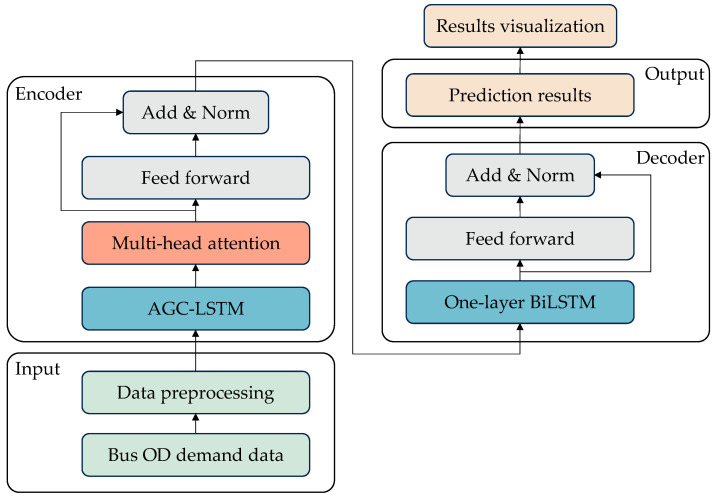
Framework of the proposed PAG-STAN model.

The PAG-STAN is a complex deep learning framework comprising four sequential components: input layer, encoder, decoder, and output layer, as illustrated in Figure 1. Each component is interconnected in the PAG-STAN framework, collectively transforming raw input data into final prediction results.

(i) The input layer primarily receives raw bus passenger flow data, including historical OD demands and their temporal information. These data are formatted as tensors and fed into the model to support subsequent feature extraction and processing.
(ii) In the encoder layer, the OD demand data is first input into the AGC-LSTM module, which captures hidden periodic spatiotemporal distribution features. The captured information is then encoded using an attention mechanism to generate corresponding representations. These representations are subsequently passed through a residual network to enhance the periodic spatiotemporal feature representations.
(iii) The decoder incorporates one-layer BiLSTMs, which serve to decompress and reconstruct the enhanced spatiotemporal representations back into a space with the same dimensions as the original OD demand data.
(iv) Finally, the output layer uses the decoded spatiotemporal features to generate short-term OD demand prediction results. The output is typically a tensor of the same dimensions as the actual OD data, representing predicted demand between stops across various time intervals.

### 2.2. AGC-LSTM

In the context of bus demand prediction, complex spatial dependencies often exist among bus stops, i.e., fluctuations in demand at one stop may influence the demand at nearby stops. To represent the spatial relationships between stops, the bus network is defined as $G=\left(V,E,A\right)$, where $V=\left\{v_{1},v_{2},\ldots ,v_{N}\right\}$ denotes the set of stops and N represents the number of stops. E denotes the set of edges. $A\in R^{N\times N}$ denotes the adjacency matrix, which represents the distance between adjacent stops.

Researchers usually adopt GCNs to effectively capture these spatial correlations [23]. The graph convolution is typically implemented through matrix operations. The core formula is as follows.

(1) $$H_{l+1}=\sigma \left({\tilde{D}}^{-1/2}\tilde{A}{\tilde{D}}^{-1/2}H_{l}W_{l}\right)$$

where $H_{l}$ denotes the node feature matrix of the *l*th layer. σ(⋅) represents an activation function. $\tilde{A}=A+I$ is the adjacency matrix with self-connections added. I is the identity matrix. $\tilde{D}$ is the degree matrix of $\tilde{A}$. $W_{l}$ is the learnable weight matrix of the *l*th layer.

**Figure 2:**
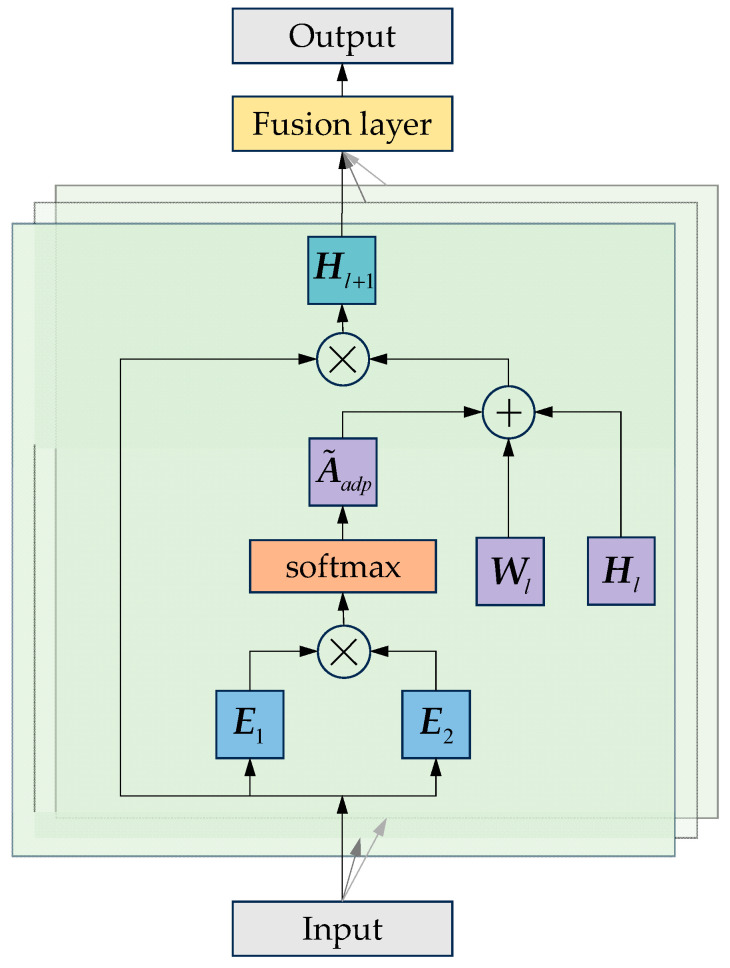
Structure of the AGCN.

Although traditional GCNs are effective at learning topological spatial features, the spatial dependencies among traffic nodes often change over time. A fixed adjacency matrix may constrain the model’s prediction performance. Therefore, this study incorporates an adaptive adjacency matrix into the GCN, forming an Adaptive Graph Convolutional Network (AGCN) [38]. Figure 2 demonstrates the structure of the AGCN.

As shown in Figure 2, before applying the adaptive adjacency mechanism, an initial static adjacency matrix is constructed to represent the basic spatial topology of the bus network. To construct the adaptive adjacency matrix, the source node $E_{1}$ and target node embeddings $E_{2}$ are randomly initialized with trainable parameters. This process can be formulated as follows.

(2) $${\tilde{A}}_{adp}=\mathrm{softmax}\left(\mathrm{ReLU}\left(E_{1}E_{2}^{T}\right)\right)$$

where the ReLU activation function is used to ignore weak connections. The softmax function is used to normalize the adaptive adjacency matrix. In our implementation, the final adjacency matrix used in each layer can be regarded as a combination of the initial static adjacency matrix and the learned adaptive adjacency matrix, allowing the model to dynamically learn hidden spatial dependencies during training. The adaptive graph convolution operation is formulated in Equation (3).

(3) $$H_{l+1}=\sigma \left(\left({\tilde{D}}^{-1/2}\tilde{A}{\tilde{D}}^{-1/2}⊕{\tilde{A}}_{adp}\right)H_{l}W_{l}\right)$$

The LSTM layer mainly functions to process time series information. It analyzes the data through its internal mechanisms of the forget gate, input gate, and output gate [39], thereby capturing temporal variation patterns.

**Figure 3:**
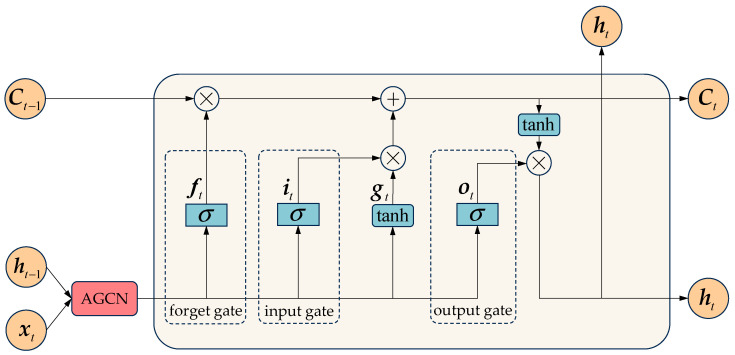
Structure of the LSTM.

Figure 3 shows the structure of an LSTM network. The adaptive graph convolutional layer first encodes the spatial information and extracts features among nodes. These features are then passed to the LSTM layer, which further processes them along the temporal dimension. The gating mechanisms in the LSTM collaborate to determine which information should be retained, updated, and output.

As shown in Figure 3, the forget gate $f_{t}$ controls how much information from the previous cell state $C_{t-1}$ should be discarded. The input $x_{t}$ and the hidden state from the previous time step $h_{t-1}$ are processed through a sigmoid function, generating value between 0 and 1. The calculation of the forget gate is given by Equation (4).

(4) $$f_{t}=\sigma \left(W_{xf}*x_{t}+W_{hf}*h_{t-1}+b_{f}\right)$$

where $W_{xf}$ and $W_{hf}$ represent the weight matrices. $b_{f}$ denotes the bias vector. ∗ denotes the adaptive graph convolution operation. σ denotes the sigmoid activation function, which regulates the flow of information. Equation (5) is formulated as follows.

(5) $$\mathrm{sigmoid}\left(x\right)=\frac{1}{1+\exp\left(-x\right)}$$

**Figure 4:**
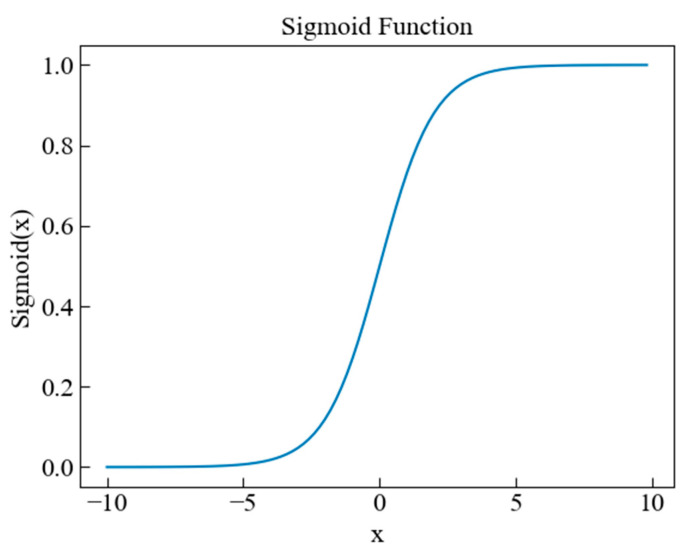
Graph of the sigmoid activation function.

As shown in Figure 4, the graph of the sigmoid function is an S-shaped curve that monotonically increases over its domain. It is symmetric about the point (0, 0.5), and its output lies within the interval (0, 1). When the output approaches 0, it implies that the information is entirely forgotten. Otherwise, when it approaches 1, the information is almost entirely retained.

The input gate $i_{t}$ determines how much new information from the candidate cell state $g_{t}$ should be added to the memory cell. The candidate state is computed using the tanh function, and then it is combined with historical information and the new candidate content to update the cell state $C_{t}$. Equations (6)–(8) describe this process as follows.

(6) $$i_{t}=\sigma \left(W_{xi}*x_{t}+W_{hi}*h_{t-1}+b_{i}\right)$$

(7) $$g_{t}=\tanh\left(W_{xg}*x_{t}+W_{hg}*h_{t-1}+b_{g}\right)$$

(8) $$C_{t}=f_{t}⊙C_{t-1}+i_{t}⊙g_{t}$$

where ⊙ denotes the Hadamard product. tanh(⋅) denotes the hyperbolic tangent activation function. $W_{xi}$, $W_{hi}$, $W_{xg}$, $W_{hg}$ represent the weight matrices. $b_{i}$ and $b_{g}$ denote the bias vectors.

The output gate $o_{t}$ determines how much information from the updated cell state $C_{t}$ should be transferred to the hidden state $h_{t}$. The gate $o_{t}$ is multiplied by the cell state transformed by the tanh function to obtain the hidden state $h_{t}$. Calculation formulas are as follows.

(9) $$o_{t}=\sigma \left(W_{xo}*x_{t}+W_{ho}*h_{t-1}+b_{o}\right)$$

(10) $$h_{t}=o_{t}⊙\tanh\left(C_{t}\right)$$

where $W_{xo}$ and $W_{ho}$ are the weight matrices. $b_{o}$ denotes the bias vector.

### 2.3. Attention Mechanism

The attention mechanism in the PAG-STAN model is implemented based on a multi-head attention architecture. Multi-head attention enables the model to compute attention weights within multiple subspaces, thereby capturing associations among different positions in the input sequence more comprehensively.

**Figure 5:**
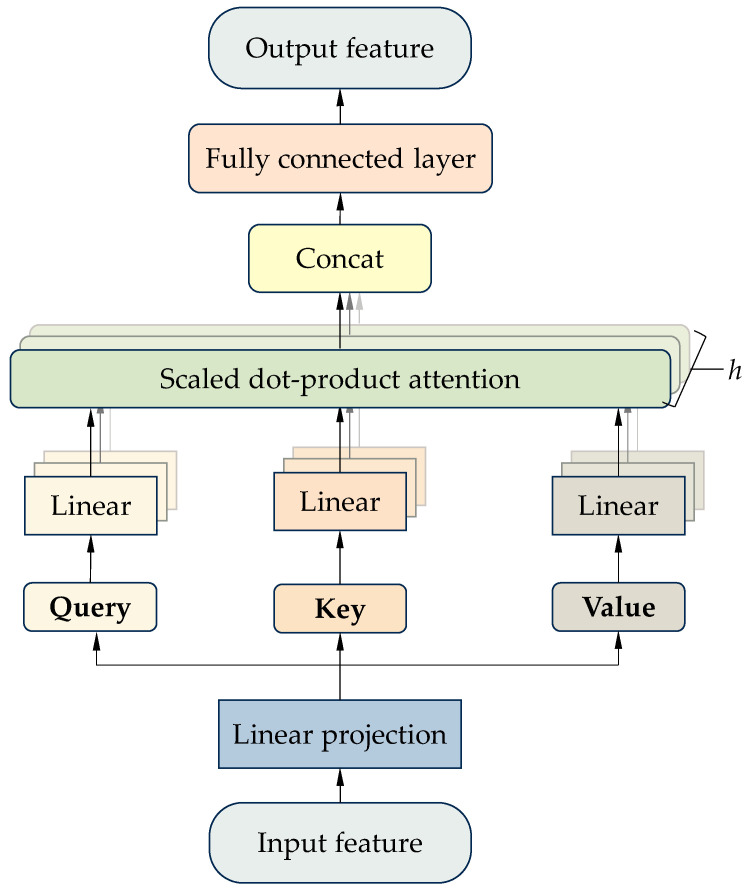
Computation process of the multi-head attention mechanism.

Figure 5 illustrates the computation process of the multi-head attention mechanism, which can be summarized in the following four steps.

Step 1: Three different linear transformation layers are applied to the input sequence to obtain the query vector (***Q***), key vector (***K***), and value vector (***V***). The query vector represents the information that needs to be focused on at the current moment, the key vector represents the feature at each position in the input sequence, and the value vector contains the actual content information of each position in the input sequence. Calculation formulas in Step 1 are as follows.

(11) $$Query=XW_{Q}$$

(12) $$Key=XW_{K}$$

(13) $$Value=XW_{V}$$

where X denotes the input sequence. $W_{Q}$, $W_{K}$ and $W_{V}$ represent the learnable weight matrices.

Step 2: The query, key, and value vectors are split into multiple attention heads, with each head corresponding to a different subspace. The dimension of each attention head is calculated using Equation (14).

(14) $$d_{k}=d_{model}/Head_{num}$$

where $d_{model}$ denotes the dimensionality of each input vector. $Head_{num}$ denotes the number of attention heads.

Step 3: Each head independently computes attention scores within its own subspace. First, the dot product of ***Q*** and ***K*** is computed and then scaled to obtain the attention scores, which represent the degree of correlation between the current position and other positions in the sequence. Second, to ensure interpretability and numerical stability, the attention scores are then normalized using the softmax function to produce attention weights. Third, the attention weights are used to perform a weighted sum of the value vectors, obtaining the output of each attention head. The formulas for Step 3 are as follows.

(15) $$\mathrm{score}\left(Q,K\right)=\frac{QK^{T}}{\sqrt{d_{k}}}$$

(16) $$Attention\;Weights=\mathrm{softmax}\left(\mathrm{score}\left(Q,K\right)\right)$$

(17) $$Output_{i}=\sum_{j=1}^{n}V_{j}\cdot Attention\;Weights_{ij}$$

where $\sqrt{d_{k}}$ denotes the scaling factor used to prevent gradient vanishing or explosion. $Output_{i}$ denotes the *i*th element in the output sequence. $Attention\;Weights_{ij}$ represents the attention weight of the *i*th element on the *j*th value vector.

Step 4: The outputs of all attention heads are concatenated and then linearly transformed using a projection matrix. The result as the final output of the multi-head attention mechanism is written as Equation (18).

(18) $$\mathrm{MultiHead}\left(Q,K,V\right)=\mathrm{Concat}\left(head_{1},\ldots ,head_{n}\right)W_{o}$$

### 2.4. Bidirectional LSTM

**Figure 6:**
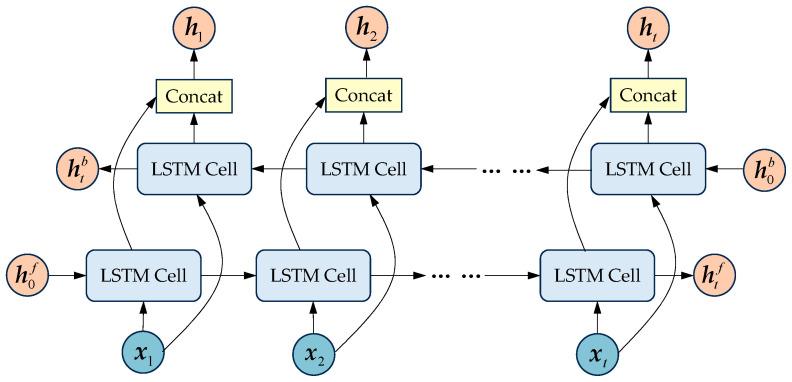
Structure of the BiLSTM.

After learning the periodic spatiotemporal distribution information in the encoder, the decoder then utilizes one-layer BiLSTMs to decode the information in both forward and backward directions. Although LSTM is effective in capturing long-term dependencies, it cannot process input sequences in reverse order, which limits its ability to handle future information. BiLSTM consists of a forward LSTM layer and a backward LSTM layer, as shown in Figure 6. These two layers process the input sequence in forward and reverse directions, respectively. The forward and backward hidden states at each time step are merged through concatenation. As a result, the final hidden state contains information from both past and future moments, enabling the model to better understand the contextual relationships within the input sequence. This process is formulated as follows.

(19) $$h_{t}^{f}=LSTM\left(x_{t},h_{t-1}^{f}\right)$$

(20) $$h_{t}^{b}=LSTM\left(x_{t},h_{t-1}^{b}\right)$$

(21) $$h_{t}=h_{t}^{f}⊕h_{t}^{b}$$

where LSTM(⋅) is the LSTM cell, $h_{t}^{f}$ is the forward hidden state, $h_{t}^{b}$ is the backward hidden state, and ⊕ denotes the concatenation operator.

## 3. Dataset

### 3.1. Data Description

**Figure 7:**
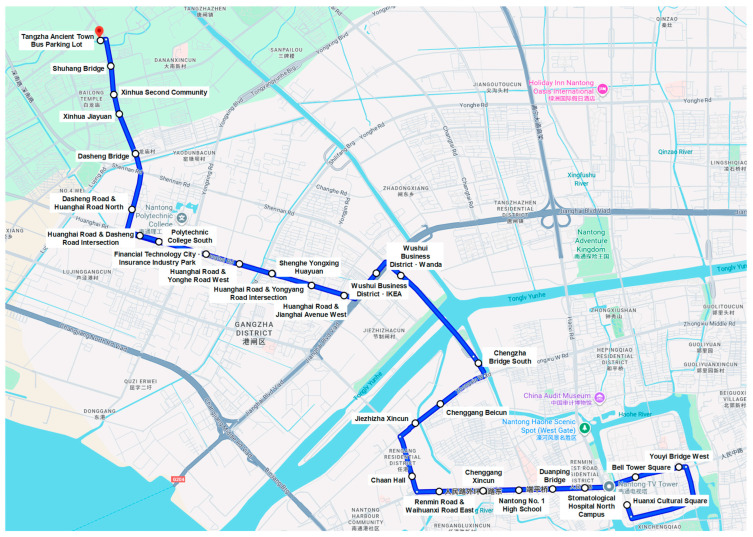
Nantong No. 1 bus route map.

In the case study, numerical experiments based on a real-world single-line bus corridor in China validate the developed framework. The No. 1 bus route of Nantong includes 27 stops. Figure 7 is the Nantong No. 1 bus route map, which is generated based on a screenshot from Google Maps with stop labels added manually. All bus stop IDs and names, the spacing between adjacent stops, as well as cumulative distance are listed in Table 2. The average spacing between adjacent stops is approximately 514 m, which aligns well with the density of Nantong’s downtown road network and pedestrian accessibility standards. The current stop configuration ensures convenient boarding and alighting within the main urban area while maintaining operational efficiency. The data is derived from bus passenger flow IC card records. Passengers are required to swipe their cards both when boarding and alighting. Our experiment uses historical OD passenger flow data in the outbound direction from 2 September to 9 September 2024. Data were collected daily from 5:00 to 21:00 at 30 min intervals. The first seven days of data are used as the training set, and the last day as the test set. 20% of the training set is reserved for validation. The description of the dataset is provided in Table 3. Table 4 presents sample data examples, i.e., passenger flow records between partial OD pairs from 7:30 to 8:00 on September 2. The dataset is indexed by timestamps, and each timestamp corresponding to a 27 × 27 OD matrix.

Based on the passenger flow variations during morning and evening peaks as well as between weekdays and weekends, the following characteristics of passenger flow can be obtained.

**Figure 8:**
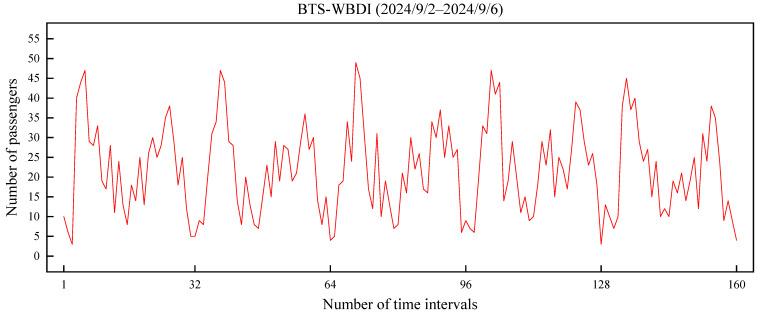
Trend of bus OD demand over time.

(i) Passenger flow significantly increases during peak hours. During morning and evening commuting periods, passenger volumes along the entire route rise sharply, with more boarding activity at all stops. Figure 8 shows the variation trend of bus OD demand from Bell Tower Square Stop to Wushui Business District—IKEA Stop between 2 and 6 September 2024. The horizontal axis represents the number of time intervals, with each interval being 30 min, and there are 32 intervals per day. The vertical axis represents the OD demand volume. It can be intuitively observed from the figure that there is a periodic fluctuation in passenger flow. Higher OD demand appears during the morning and evening peak hours, whereas the demand is relatively lower during off-peak hours.
(ii) Passenger flow is influenced by temporal factors. During off-peak hours, such as weekday mornings, afternoons, and evenings, passenger flow is relatively low. On weekends and holidays, with fewer commuters, the overall passenger volume generally decreases. Taking the trip from Bell Tower Square Stop to Wushui Business District—IKEA Stop as an example, the average daily OD demand is 728 passengers on weekdays and 415 passengers on weekends, representing a decrease of approximately 43% on weekends compared to weekdays.

### 3.2. Experimental Results

#### 3.2.1. Normalization Details

The data preprocessing procedures are as follows.

Step 1: Remove outliers from the dataset and fill in missing values.

Step 2: Convert the OD passenger flow data observed at each time interval into 27 × 27 OD matrices, with corresponding timestamp information recorded.

Step 3: Apply Min-Max Scaling to normalize the OD data into the range of [0, 1] to ensure proportionate scaling. This improves model stability and training speed. The normalization formula is written as follows.

(22) $$X_{norm}=\frac{X-X_{min}}{X_{max}-X_{min}}$$

where $X_{norm}$ denotes the normalized value. X denotes the original value. $X_{max}$ and $X_{min}$ represent the maximum and minimum values in the original dataset, respectively.

#### 3.2.2. Experimental Environment

All models in this paper are implemented with PyTorch on a personal computer with Intel Core i7-1260P CPU @ 2.10 GHz and 16 GB RAM. The environment configuration is summarized in Table 5.

#### 3.2.3. Hyperparameter Optimization

**Figure 9:**
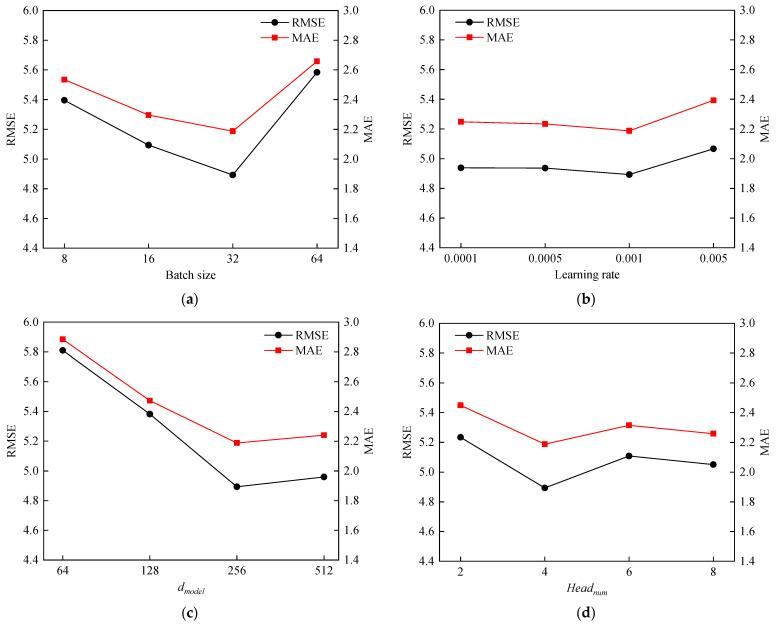
Hyperparameter tuning results. (**a**) Impact of batch size on PAG-STAN. (**b**) Impact of learning rate on PAG-STAN. (**c**) Impact of $d_{\mathrm{mod}el}$ on PAG-STAN. (**d**) Impact of $Head_{num}$ on PAG-STAN.

To determine the optimal hyperparameters, this paper elaborates on the impact of four hyperparameters of PAG-STAN on the model performance. These hyperparameters are the training batch size, learning rate, dimension of feature vectors $d_{\mathrm{mod}el}$, and number of attention heads $Head_{num}$. Specifically, we set the section for the batch size to (8, 16, 32, 64), the section for the learning rate to (0.0001, 0.0005, 0.001, 0.005), the section for $d_{\mathrm{mod}el}$ to (64, 128, 256, 512), and the section for $Head_{num}$ to (2, 4, 6, 8). The impacts of the above four hyperparameters on model performance are shown in Figure 9.

As shown in Figure 9a, with the batch size increases, both the root mean square error (RMSE) and the mean absolute error (MAE) gradually decrease. However, when the batch size exceeds 32, the prediction performance shows a significant decline. Therefore, we set the training batch size to 32. Similarly, in Figure 9b–d, the hyperparameters corresponding to the simultaneous minimum values of RMSE and MAE are regarded as the optimal hyperparameters. Therefore, we set the learning rate to 0.001, $d_{\mathrm{mod}el}$ to 256, and $Head_{num}$ to 4. For other parameter settings, the number of training epochs was set to 500 according to the early stopping strategy, the dropout rate is 0.1, and the Adam optimizer is used to update the model parameters. Table 6 is a summary of the parameter values of PAG-STAN.

#### 3.2.4. Loss Function

Before training the model, it is essential to define the loss function. This ensures that the model parameters are continuously optimized during training, thereby improving the accuracy of prediction results. The role of the loss function is to quantify the difference between the predicted OD demand and the actual observed values. It is minimized iteratively until convergence. Deep learning models often function as “black boxes”. Since there is a strong quantitative relationship between OD demand data and inbound passenger flow, which reflects the physical law in the process of OD distribution change, incorporating this relationship into the loss function can enhance model interpretability. Therefore, embedding the law of passenger flow into the loss function can effectively guide model training. Additionally, zero values are used to fill in missing values when constructing the OD matrix. However, these zero values may lead to a decline in prediction performance. Therefore, a masking operation is introduced in the loss function to avoid training errors caused by the padded zero values. The masked physics-guided (MPG) loss function [1] is written in Equation (23) as follows.

(23) $$\begin{array}{l} MPG-loss=W_{OD}\cdot \frac{1}{{\left(N\times K\right)}_{no\_mask}}\sum_{i\in N}\sum_{j\in K}{\left(y_{ij}-{\hat{y}}_{ij}\right)}^{2}\\ \;\;\;\;\;\;\;\;\;\;\;\;\;\;\;\;\;\;\;\;\;\;\;\;\;\;+W_{in}\cdot \frac{1}{N}\sum_{i\in N}{\left(\sum_{j\in K}y_{ij}-P_{i}^{in}\right)}^{2} \end{array}$$

where $W_{OD}$ and $W_{in}$ represent the weights of different terms in the loss function, which can dynamically adjust the influence of different types of information during model training. ${\left(N\times K\right)}_{no\_mask}$ denotes the number of unmasked OD pairs involved in backpropagation. $y_{ij}$ refers to the actual OD demand between stop $s_{i}$ and stop $s_{j}$. ${\hat{y}}_{ij}$ is the predicted value. $P_{i}^{in}$ signifies the inbound passenger flow at stop i.

The MPG loss function is a weighted combination of a data-driven term and a physics-constrained term. The former evaluates the model’s capability to learn the OD distribution features, while the latter evaluates its ability to learn physical laws. The core contribution of the physics-guided loss term is to serve as a transportation theory-based regularization constraint. Specifically, it penalizes predictions that violate fundamental physical principles governing passenger flow changes. The passenger flow conservation law dictates that the total predicted outbound passenger flow from a station should approximately match the total inbound flow entering other stops. By introducing the physics-constrained term, the model is penalized whenever its predicted OD matrix leads to inconsistencies between total departures and total arrivals across the network, thereby preventing physically implausible spikes or drops in passenger demand. In essence, the physics prior prevents the purely data-driven model from overfitting or generating predictions that contradict well-established physical laws, thereby increasing the trustworthiness, interpretability, and domain relevance of the PAG-STAN outputs.

#### 3.2.5. Model Training

**Figure 10:**
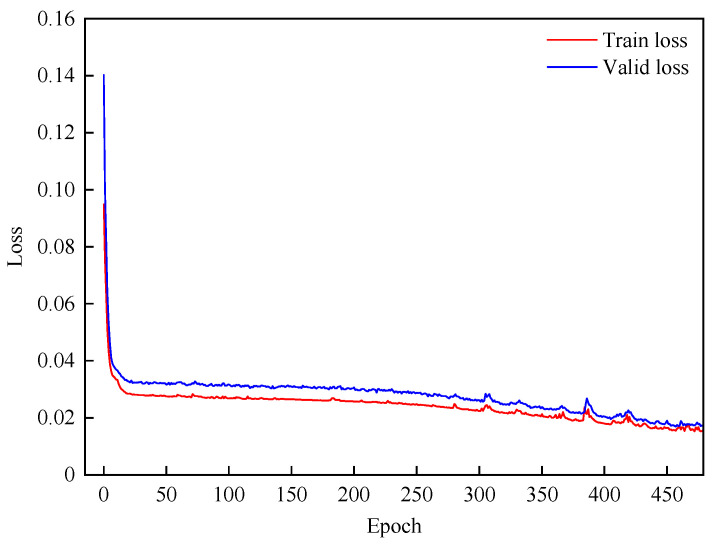
Training and validation loss curves of PAG-STAN.

During model training, the early stopping technique is used to prevent overfitting. The patience value of 20 is adopted, meaning that training stops when the validation loss does not decrease for 20 consecutive epochs. Before training is halted, the variations in training loss and validation loss are shown in Figure 10.

By comparing their trends, it can be observed that as the training error decreases, the validation error does not rebound, indicating that overfitting does not occur. Specifically, Figure 10 shows that both training and validation losses gradually converge after about the 450th training epoch. The validation loss stabilizes around the 480th epoch and becomes close to the training loss.

#### 3.2.6. Prediction Results and Analysis

**Figure 11:**
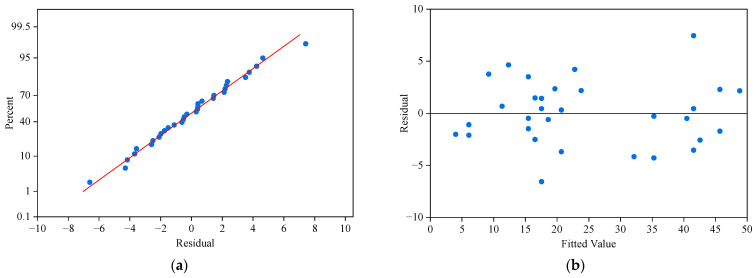

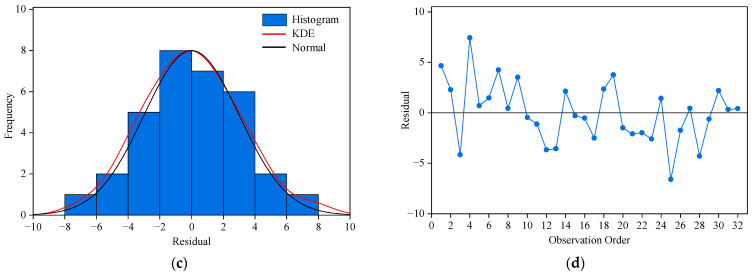
Residual plots for result. (**a**) Normal probability plot of the residuals. (**b**) Residuals versus the fitted values. (**c**) Histogram plus estimated density. (**d**) Residuals versus the order of the data.

By comparing the actual and predicted OD demand from Chenggang Xincun Stop to Huanghai Road & Yongyang Road Intersection Stop on 9 September 2024, we plotted a residual plot of the actual and predicted values, as shown in Figure 11.

Figure 11 consists of four subgraphs. Figure 11a is the normal probability plot of residuals, where the points are roughly distributed along a straight line, confirming that the residuals approximately follow a normal distribution. Figure 11b is the scatter plot of residuals versus fitted values. The residuals are randomly distributed without an obvious trend, indicating that the model has no systematic bias. Figure 11c shows the residual histogram and kernel density estimation curve, with the black curve representing the normal distribution curve. This graph reveals that the residuals are approximately normally distributed and concentrated around zero, suggesting a reasonable error distribution. Figure 11d is the plot of residuals changing with the observation order. There is no obvious temporal correlation among residuals, indicating good stability of the model.

Although the OD demand from Chenggang Xincun Stop to Huanghai Road & Yongyang Road Intersection Stop exhibits irregular fluctuations, the model can effectively learn the spatiotemporal distribution of OD demand from historical OD data. By leveraging historical daily OD demand data, PAG-STAN successfully captures the periodic patterns in the demand data, thereby achieving a good fit between the predicted results and the actual demand. These results demonstrate that PAG-STAN exhibits strong prediction performance.

**Figure 12:**
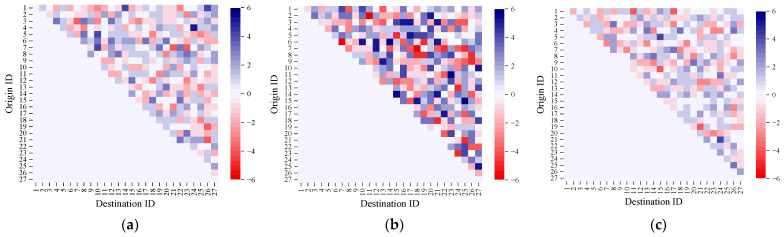
Real and predicted OD demand differences during peak and off-peak hours. (**a**) Difference heatmap 7:30–8:00. (**b**) Difference heatmap 12:30–13:00. (**c**) Difference heatmap 17:30–18:00.

Meanwhile, we utilize heatmaps to visually present the differences between the actual OD passenger flow matrices and the predicted results. Figure 12 shows the actual and predicted OD demand differences for all OD pairs during the morning peak period (7:30–8:00), noon off-peak period (12:30–13:00), and evening peak period (17:30–18:00) on 9 September 2024. In the heatmaps, the vertical axis represents the boarding stops, while the horizontal axis represents the alighting stops. The shade of the color blocks reflects the magnitude of passenger demand differences between two stops. A darker color indicates a greater demand difference.

It can be observed that the accuracy of OD demand prediction varies across different time periods, with the explanations as follows.

(i) During peak hours, passenger flow is mainly for commuting, with clear travel purposes, concentrated origins and destinations, large volume, and strong regularity. The AGC-LSTM module can effectively capture the spatiotemporal correlation of OD demand, and the attention mechanism is also more likely to focus on the characteristics of passenger flow during peak hours, resulting in small prediction errors.
(ii) During off-peak hours, the difference between predicted values and real values is significantly larger than that in peak hours. Noon passenger flow is mainly for non-commuting trips, with small volume, scattered travel purposes, and strong randomness, which lead to weakened spatiotemporal correlation and a lack of stable patterns in passenger flow demand. As a result, the model has limited ability to learn such aperiodic characteristics, leading to larger deviations.

### 3.3. Comparison with State-of-the-Art Methods

#### 3.3.1. Evaluation Metrics

To compare the prediction quality, the sophisticated evaluation metrics are utilized. These include the root mean square error (RMSE), mean absolute error (MAE), weighted mean absolute percentage error (WMAPE), and the coefficient of determination (R^2^), as formulated in Equations (24)–(27).

(24) $$RMSE=\sqrt{\frac{1}{{\left(N\times K\right)}_{no\_mask}}\sum_{i\in N}\sum_{j\in K}{\left(y_{ij}-{\hat{y}}_{ij}\right)}^{2}}$$

(25) $$MAE=\frac{1}{{\left(N\times K\right)}_{no\_mask}}\sum_{i\in N}\sum_{j\in K}\left|y_{ij}-{\hat{y}}_{ij}\right|$$

(26) $$WMAPE=\frac{\sum_{ij}\left|y_{ij}-{\hat{y}}_{ij}\right|}{\sum_{ij}y_{ij}}$$

(27) $$R^{2}=1-\frac{\sum_{ij}{\left(y_{ij}-{\hat{y}}_{ij}\right)}^{2}}{\sum_{ij}{\left(y_{ij}-\bar{y}\right)}^{2}}$$

#### 3.3.2. Baseline Models

In order to evaluate the effectiveness of the proposed model, five commonly used prediction models and two modules from the PAG-STAN are compared with PAG-STAN in this section. For the seven comparative models, we set the number of training epochs to 500 and the dropout rate to 0.1. All models use the Adam optimizer to update their parameters. The early stopping technique is applied to prevent overfitting, with the patience value set to 20. The hyperparameter tuning process for all models is conducted under the same settings, where the sections for batch size, learning rate, number of neurons, and number of attention heads are consistent with those of the PAG-STAN model. Other parameter settings of these seven baseline models are given as follows.

- **2D CNN:** 2D CNN extracts rich feature information through convolution operations. It consists of two 2D CNN layers with 3 × 3 filters and a fully connected layer with 256 neurons. The learning rate is 0.0005. The batch size is 16.
- **LSTM:** LSTM effectively captures key features in time series. Two fully connected LSTM layers are used to predict future OD demand, with a hidden state dimension set to 256. The learning rate is 0.0005. The batch size is 16.
- **STGCN:** STGCN [40] utilizes graph convolution to capture spatial dependencies and temporal convolution to model temporal features. This model stacks multiple ST-Conv blocks. In our study, we set the graph convolution kernel size and temporal convolution kernel size to 3. The learning rate is 0.001. The batch size is 32.
- **BiLSTM:** We use the bidirectional LSTM module from the PAG-STAN decoder as an independent baseline model.
- **ConvLSTM:** ConvLSTM [41] replaces the fully connected layers in LSTM with convolutional layers, enabling it to capture spatial and temporal features simultaneously. The convolution kernel size is 3 × 3. The learning rate is 0.001. The batch size is 32. Other model configurations are consistent with those of LSTM.
- **AGC-LSTM:** We extract the adaptive graph convolutional LSTM module from the PAG-STAN encoder as an independent baseline model.
- **Transformer:** Transformer [28] is an attention-based model that learns attention weights from sequential data using multi-head attention mechanisms. The learning rate is 0.001. The batch size is 32. The number of heads was set to 4, and the feature vector dimension was set to 256.

#### 3.3.3. Model Comparisons

Table 7 presents the experimental results. It can be observed that the proposed PAG-STAN model achieves the best prediction performance, followed by the Transformer model. The RMSE of PAG-STAN is 4.8927, which is 6.19% lower than that of Transformer. Its MAE is 2.1878, 6.59% lower than Transformer’s MAE. Its WMAPE is 21.39%, 8.20% lower than Transformer’s. Last, its R^2^ reaches 0.9755, which is 1.13% higher than Transformer’s. Furthermore, to validate the necessity of the core components within the PAG-STAN framework, we specifically benchmarked AGC-LSTM and BiLSTM as standalone models. As shown in Table 7, the prediction performance of the isolated AGC-LSTM model and the isolated BiLSTM model is weaker than that of the integrated PAG-STAN framework. The performance gap clearly demonstrates the necessity and effectiveness of integrating AGC-LSTM, BiLSTM, and the multi-head attention mechanism, thereby indirectly confirming that each designed module plays a crucial role in improving the prediction accuracy of PAG-STAN.

In addition to prediction accuracy, we evaluated the model’s computational efficiency. As summarized in Table 7, the average training time per epoch for PAG-STAN is 22.19 s, and the inference time for a single prediction is 1.12 s, which are within acceptable ranges for short-term prediction. Although the model incorporates multiple components, its complexity remains feasible for deployment on standard computing platforms. These results indicate that the proposed framework achieves a favorable balance between accuracy and computational efficiency.

**Figure 13:**
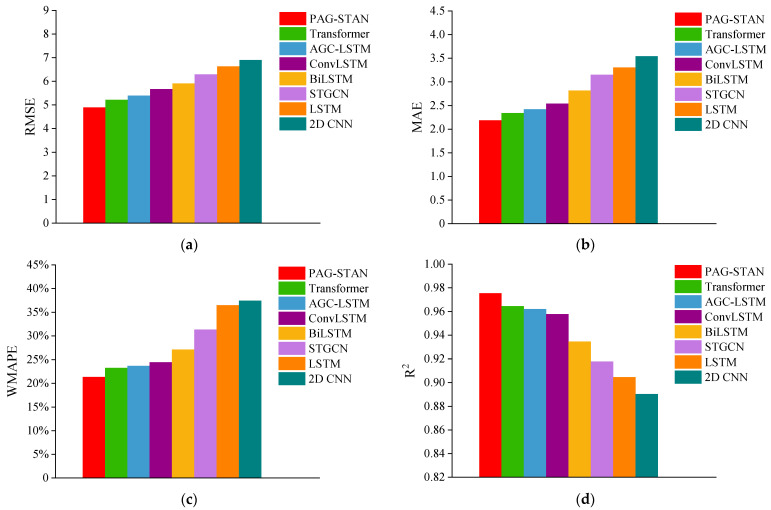
Comparison of performances of different models. (**a**) Comparison of RMSE. (**b**) Comparison of MAE. (**c**) Comparison of WMAPE. (**d**) Comparison of R^2^.

Moreover, we utilize bar charts to intuitively compare the performance of each model in the prediction task, as shown in Figure 13. For the error metrics (RMSE, MAE, WMAPE), lower values indicate higher prediction accuracy. PAG-STAN performs best in all three metrics with the lowest values. For the fitting metric R^2^, value closer to 1 indicate better data fitting. PAG-STAN achieves the highest R^2^, showing the best fitting performance. Overall, PAG-STAN outperforms the other seven models in both prediction accuracy and data fitting degree.

**Figure 14:**
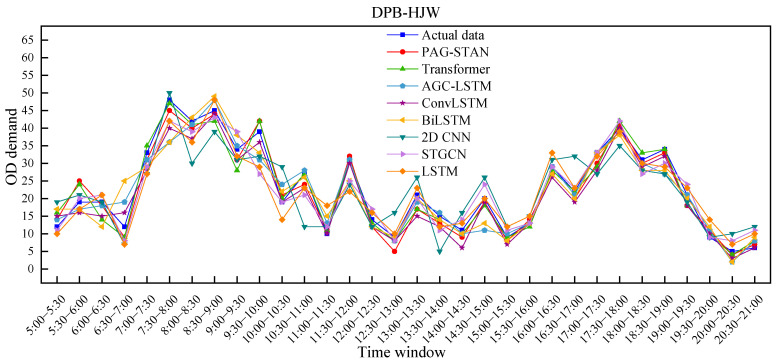
Comparison of prediction results of different models.

Figure 14 illustrates the comparison between actual and predicted OD demand for each model on 9 September 2024, from Duanping Bridge Stop to Huanghai Road & Jianghai Avenue West Stop. Actual values are marked with squares, while predicted values of the proposed model are marked with circles. As shown in Figure 14, compared to other methods, the PAG-STAN model more accurately captures the peaks and troughs of OD demand fluctuations throughout the day. The following specific conclusions can be obtained.

(i) The performance of different models across various time intervals is generally consistent. In particular, PAG-STAN performs better than the others during both peak and off-peak periods, indicating its stability.
(ii) When OD demand reaches peaks or troughs, the performance gap among models becomes more pronounced. In such cases, the PAG-STAN model exhibits its superior ability to capture the passenger flow fluctuations.
(iii) When OD demand nonlinear fluctuation, performance varies across all models. Nonetheless, PAG-STAN still achieves the best predictions in this situation, demonstrating its strong adaptability.

## 4. Conclusions

This paper focuses on the short-term OD demand prediction problem in bus travel. Our study demonstrates that even when trained on small-scale datasets, the improved PAG-STAN model can effectively capture the essential spatiotemporal patterns and achieve reliable prediction accuracy. Conducting large-scale experiments would result in disproportionately high computational costs, with only marginal improvements in precision. Therefore, focusing on small-scale data is consistent with the situation of limited data availability in real-world bus operations, while also ensuring the practicality of the proposed approach. The main contributions are as follows.

(i) This paper systematically summarizes the shortcomings of existing research on traffic/passenger demand prediction, including the limitations of traditional statistical models and machine learning models, the scarcity of studies specifically addressing bus OD prediction, and the lack of deep learning-based prediction models developed for small-scale bus datasets.
(ii) To address these issues, this paper proposes an improved PAG-STAN framework. The framework simplifies the input into daily OD data, enabling the model to extract daily periodic features. A multi-head attention module is embedded in the encoder to enhance the model’s feature learning and representation capabilities, while one-layer bidirectional LSTMs are adopted in the decoder to reduce the risk of overfitting under the condition of small-scale training set.
(iii) Experiments on a small-scale Nantong bus OD demand dataset demonstrate that the PAG-STAN model outperforms other baseline models in terms of applicability, stability, and prediction accuracy.

Although our study has achieved certain research outcomes, due to limitations in the research scope, there are still several limitations. Future research can be extended in the following directions.

(i) In future research, the focus can be placed on expanding the scale of the dataset by collecting OD passenger flow data from multiple bus routes over longer time spans and validating the model across different urban environments, so as to more comprehensively evaluate its robustness and transferability. Meanwhile, since the experimental data used in this study cover an earlier period from 2 September to 9 September 2024, it is necessary to acquire and utilize more recent datasets in future work to enhance the reliability and timeliness of the model evaluation.
(ii) There are many factors influencing bus OD demand. It is challenging to accurately capture dynamic patterns using only a single type of bus data. Future studies can incorporate multidimensional data such as weather conditions, holiday travel demands, etc., to build more robust and reliable prediction models.
(iii) In future research, to improve prediction accuracy and stability during off-peak hours, we will also focus on enhancing the model’s capability to capture irregular and low-demand patterns, which may be achieved by refining the model architecture or incorporating additional contextual features.
(iv) The significance of bus OD demand prediction lies in its potential to help optimize and manage public transportation systems. Future work will explore the integration of the proposed model with manual or rule-based scheduling strategies to evaluate its potential for supporting real-world operational decision-making in the urban bus system.

## Figures and Tables

**Table 1:** Comparison of relevant studies on deep learning-based prediction approaches.

References	Research Question	Input Data	Model(s)	Attention Mechanism(s)	Objective(s)	Compared with the Suboptimal Model
Zhao et al. [32] (2019)	Traffic prediction based on urban road networks	Real-world traffic dataset	GCN, GRU	None	To predict traffic flow in urban road networks	The accuracy was improved by 4.40%
Zheng et al. [33] (2020)	Short-term traffic flow prediction	Real traffic flow data	Conv-LSTM, BiLSTM	Self-attention	To effectively capture complex nonlinear characteristics of traffic flow	MAE, MAPE, and RMSE were reduced by 3.55%, 8.06%, and 4.15%
Zhang et al. [22] (2020)	Short-term metro passenger flow prediction	Smart card data of Beijing Metro	3D CNN, GCN	None	To capture periodic features of metro passenger flow	RMSE, MAE, and WMAPE were reduced by 9.26%, 5.60%, and 2.80%
Ke et al. [36] (2021)	Short-term ride-hailing demand prediction	For-hire-vehicles datasets in Manhattan, New York City	RMGC, LSTM	None	To predict ride-hailing demand of various OD pairs	RMSE, MAE, and MAPE were reduced by 4.24%, 3.90%, and 5.00%
Huang et al. [37] (2022)	Short-term ride-hailing demand prediction	Real-world dataset during COVID-19 in Beijing	LSTM	Multi-head spatial attention, bidirectional attention	To predict the demand of urban ride-hailing during the epidemic period	RMSE, MAE, and MAPE were reduced by 4.03%, 2.30%, and 4.82%
Zhang et al. [20] (2023)	Short-term metro passenger flow prediction	Smart card data of Beijing metro and Xiamen metro	GCN, 3D CNN, ResNet	Multi-head attention	To predict systematic short-term passenger flow in urban rail transit	RMSE, MAE, and WMAPE were reduced by 10.35%, 7.43%, and 4.30%
Yang et al. [28] (2023)	Short-term passenger flow prediction of multiple transportation modes	Original passenger flow data of three transportation modes in selected areas of Beijing	ResNet, Transformer	Multi-head attention	To predict short-term inflow for multiple transportation modes	RMSE, MAE, and WMAPE were reduced by 1.43%, 4.70%, and 4.71%
Lv et al. [29] (2024)	Metro OD passenger flow prediction	Geographic information data, operation data, and smart card data of Shenzhen Metro	PSAM-CNN	Channel attention, spatial attention	To accurately predict OD passenger flows causing urban metro oversaturation	RMSE, MAE, and MAPE were reduced by 1.24%, 3.89%, and 8.88%
Xia et al. [21] (2024)	Traffic flow prediction	Three real-world traffic datasets	GCN, RNN, LSTM	Multi-head attention	To improve the accuracy of traffic flow prediction	RMSE, MAE, and MAPE were reduced by 7.00%, 15.70%, and 6.20%
This study	Bus short-term OD demand prediction	Nantong small-scale bus OD passenger flow dataset	AGC-LSTM, BiLSTM	Multi-head attention	To accurately predict short-term bus OD demand based on a small dataset	RMSE, MAE, and WMAPE were reduced by 6.19%, 6.59%, and 8.20%, and R^2^ was increased by 1.13%

**Table 2:** Relevant information of all bus stops.

Stop IDs	Stop Names	Stop Spacing (m)	Cumulative Distance (m)
1	Huanxi Cultural Square		
		1500	1500
2	Youyi Bridge West		
		406	1906
3	Bell Tower Square		
		607	2513
4	Stomatological Hospital North Campus		
		366	2879
5	Duanping Bridge		
		427	3306
6	Nantong No. 1 High School		
		380	3686
7	Chenggang Xincun		
		510	4196
8	Renmin Road & Waihuanxi Road East		
		424	4620
9	Chaan Hall		
		589	5209
10	Jiezhizha Xincun		
		378	5587
11	Chenggang Beicun		
		833	6420
12	Chengzha Bridge South		
		1200	7620
13	Wushui Business District—Wanda		
		408	8028
14	Wushui Business District—IKEA		
		686	8714
15	Huanghai Road & Jianghai Avenue West		
		330	9044
16	Huanghai Road & Yongyang Road Intersection		
		260	9304
17	Shenghe Yongxing Huayuan		
		382	9686
18	Huanghai Road & Yonghe Road West		
		523	10,209
19	Financial Technology City—Insurance Industry Park		
		503	10,712
20	Polytechnic College South		
		194	10,906
21	Huanghai Road & Dasheng Road Intersection		
		418	11,324
22	Dasheng Road & Huanghai Road North		
		677	12,001
23	Dasheng Bridge		
		516	12,517
24	Xinhua Jiayuan		
		155	12,672
25	Xinhua Second Community		
		448	13,120
26	Shuhang Bridge		
		248	13,368
27	Tangzha Ancient Town Bus Parking Lot		
	

**Table 3:** Description of dataset.

Description	Nantong Small-Scale Bus OD Dataset
Date	2 September 2024–9 September 2024
Time	5:00 to 21:00
Direction	Upward
Day number	8
Stop number	27
Matrix dimension	27 × 27
Time interval	30 min
Matrix number in a day	32
Train timespan	2 September 2024–8 September 2024
Validation timespan	20% of the training set
Test timespan	9 September 2024

**Table 4:** Sample data examples.

	D	1	2	3	4	…	24	25	26	27
O										
1		0	13	10	16	…	7	0	1	4
2		0	0	15	23	…	5	2	5	0
3		0	0	0	9	…	1	3	1	4
4		0	0	0	0	…	4	2	3	2
⋮		⋮	⋮	⋮	⋮	⋱	⋮	⋮	⋮	⋮
24		0	0	0	0	…	0	4	6	1
25		0	0	0	0	…	0	0	2	2
26		0	0	0	0	…	0	0	0	1
27		0	0	0	0	…	0	0	0	0

**Table 5:** Experimental environment configuration.

Software/Library	Version
Python	3.12.7
Pandas	2.2.3
PyTorch	2.5.1
Numpy	2.0.1
Matplotlib	3.10.0

**Table 6:** Parameters of PAG-STAN.

Parameters	Values
Batch size	32
Learning rate	0.001
Epoch	500
Dropout	0.1
Optimizer	Adam
$d_{\mathrm{mod}el}$	256
$Head_{num}$	4

**Table 7:** Comparison of experimental results.

Model	RMSE	MAE	WMAPE	R^2^	Training Time Per Epoch (s)	Inference Time (s)
PAG-STAN	**4.8927**	**2.1878**	**21.39%**	**0.9755**	**22.19**	**1.12**
Transformer	5.2153	2.3421	23.30%	0.9646	16.75	0.57
AGC-LSTM	5.3949	2.4208	23.69%	0.9620	15.21	0.39
ConvLSTM	5.6688	2.5414	24.49%	0.9579	16.64	0.31
BiLSTM	5.9104	2.8168	27.10%	0.9345	15.47	0.26
STGCN	6.2993	3.1503	31.34%	0.9178	16.60	0.48
LSTM	6.6318	3.3058	36.50%	0.9047	15.43	0.27
2D CNN	6.9026	3.5423	37.45%	0.8903	15.24	0.36

The optimal and suboptimal prediction performances are highlighted in bold and underlined.

## Data Availability

The original contributions presented in this study are included in the article. Further inquiries can be directed to the corresponding author.

## References

[1] Saif M.A., Zefreh M.M., Török Á. (2019). Public Transport Accessibility: A Literature Review. Period. Polytech. Transp. Eng..

[2] Mohammed M., Oke J. (2023). Origin–Destination Inference in Public Transportation Systems: A Comprehensive Review. Int. J. Transp. Sci. Technol..

[3] Lv Y., Duan Y., Kang W., Li Z., Wang F.-Y. (2014). Traffic Flow Prediction with Big Data: A Deep Learning Approach. IEEE Trans. Intell. Transp. Syst..

[4] Fan Q., Yu C., Zuo J. (2025). Predicting Urban Rail Transit Network Origin-Destination Matrix under Operational Incidents with Deep Counterfactual Inference. Appl. Sci..

[5] Zhang S., Zhang J., Yang L., Chen F., Li S., Gao Z. (2024). Physics Guided Deep Learning-Based Model for Short-Term Origin-Destination Demand Prediction in Urban Rail Transit Systems Under Pandemic. Engineering.

[6] Smith B.L., Demetsky M.J. (1997). Traffic Flow Forecasting: Comparison of Modeling Approaches. J. Transp. Eng..

[7] Lee S., Fambro D.B. (1999). Application of Subset Autoregressive Integrated Moving Average Model for Short-Term Freeway Traffic Volume Forecasting. Transp. Res. Rec..

[8] Tan M., Wong S., Xu J., Guan Z., Zhang P. (2009). An Aggregation Approach to Short-Term Traffic Flow Prediction. IEEE Trans. Intell. Transp. Syst..

[9] Williams B.M., Hoel L.A. (2003). Modeling and Forecasting Vehicular Traffic Flow as a Seasonal ARIMA Process: Theoretical Basis and Empirical Results. J. Transp. Eng..

[10] Emami A., Sarvi M., Bagloee A.S. (2019). Using Kalman Filter Algorithm for Short-Term Traffic Flow Prediction in a Connected Vehicle Environment. J. Mod. Transp..

[11] Pan Y.A., Guo J., Chen Y., Cheng Q., Li W., Liu Y. (2024). A Fundamental Diagram Based Hybrid Framework for Traffic Flow Estimation and Prediction by Combining a Markovian Model with Deep Learning. Expert Syst. Appl..

[12] Zhao Y., Ren L., Ma Z., Jiang X. (2020). A Novel Three-Stage Framework for Prioritizing and Selecting Feature Variables for Short-Term Metro Passenger Flow Prediction. Proceedings of the Transportation Research Board 99th Annual Meeting.

[13] Wang D., Zhang Q., Wu S., Li X., Wang R. (2016). Traffic Flow Forecast with Urban Transport Network. Proceedings of the 2016 IEEE International Conference on Intelligent Transportation Engineering.

[14] Kang L., Hu G., Huang H., Lu W., Liu L. (2020). Urban Traffic Travel Time Short-Term Prediction Model Based on Spatio-Temporal Feature Extraction. J. Adv. Transp..

[15] Hong W. (2011). Traffic Flow Forecasting by Seasonal SVR with Chaotic Simulated Annealing Algorithm. Neurocomputing.

[16] Cai P., Wang Y., Lu G., Chen P., Ding C., Sun J. (2016). A Spatiotemporal Correlative k-Nearest Neighbor Model for Short-Term Traffic Multistep Forecasting. Transp. Res. Part C.

[17] Chen X., Wu S., Shi C., Huang Y., Yang Y., Ke R., Zhao J. (2020). Sensing Data Supported Traffic Flow Prediction via Denoising Schemes and ANN: A Comparison. IEEE Sens. J..

[18] Raskar C., Nema S. (2022). Metaheuristic Enabled Modified Hidden Markov Model for Traffic Flow Prediction. Comput. Netw..

[19] Jin J., Wang Y.H., Li M. (2013). Prediction of the Metro Section Passenger Flow Based on Time-Space Characteristic. Appl. Mech. Mater..

[20] Zhang X., Wang C., Chen J., Chen D. (2023). A Deep Neural Network Model with GCN and 3D Convolutional Network for Short-Term Metro Passenger Flow Forecasting. IET Intell. Transp. Syst..

[21] Xia Z., Zhang Y., Yang J., Xie L. (2024). Dynamic Spatial–Temporal Graph Convolutional Recurrent Networks for Traffic Flow Forecasting. Expert Syst. Appl..

[22] Zhang J., Chen F., Guo Y., Li X. (2020). Multi-Graph Convolutional Network for Short-Term Passenger Flow Forecasting in Urban Rail Transit. IET Intell. Transp. Syst..

[23] Shanthappa K.N., Mulangi H.R., Manjunath M.H. (2024). Origin-Destination Demand Prediction of Public Transit Using Graph Convolutional Neural Network. Case Stud. Transp. Policy.

[24] Lu X., Ma C., Qiao Y. (2021). Short-Term Demand Forecasting for Online Car-Hailing Using ConvLSTM Networks. Phys. A.

[25] Zhang Q., Li C., Su F., Li Y. (2023). Spatiotemporal Residual Graph Attention Network for Traffic Flow Forecasting. IEEE Internet Things J..

[26] He Y., Li L., Zhu X., Tsui K.L. (2022). Multi-Graph Convolutional-Recurrent Neural Network (MGC-RNN) for Short-Term Forecasting of Transit Passenger Flow. IEEE Trans. Intell. Transp. Syst..

[27] Zhan S., Cai Y., Xiu C., Zuo D., Wang D., Wong S.C. (2024). Parallel Framework of a Multi-Graph Convolutional Network and Gated Recurrent Unit for Spatial–Temporal Metro Passenger Flow Prediction. Expert Syst. Appl..

[28] Yang Y., Zhang J., Yang L., Yang Y., Li X., Gao Z. (2023). Short-Term Passenger Flow Prediction for Multi-Traffic Modes: A Transformer and Residual Network Based Multi-Task Learning Method. Inf. Sci..

[29] Lv S., Wang K., Yang H., Wang P. (2024). An Origin–Destination Passenger Flow Prediction System Based on Convolutional Neural Network and Passenger Source-Based Attention Mechanism. Expert Syst. Appl..

[30] Chen C., Liu Y., Chen L., Zhang C. (2023). Bidirectional Spatial-Temporal Adaptive Transformer for Urban Traffic Flow Forecasting. IEEE Trans. Neural Netw. Learn. Syst..

[31] Chu K.F., Lam A.Y.S., Li V.O.K. (2019). Deep Multi-Scale Convolutional LSTM Network for Travel Demand and Origin-Destination Predictions. IEEE Trans. Intell. Transp. Syst..

[32] Zhao L., Song Y., Zhang C., Liu Y., Wang P., Lin T., Deng M., Li H. (2019). T-GCN: A Temporal Graph Convolutional Network for Traffic Prediction. IEEE Trans. Intell. Transp. Syst..

[33] Zheng H., Lin F., Feng X., Chen Y. (2020). A Hybrid Deep Learning Model with Attention-Based Conv-LSTM Networks for Short-Term Traffic Flow Prediction. IEEE Trans. Intell. Transp. Syst..

[34] Noursalehi P., Koutsopoulos H.N., Zhao J. (2021). Dynamic Origin-Destination Prediction in Urban Rail Systems: A Multi-Resolution Spatio-Temporal Deep Learning Approach. IEEE Trans. Intell. Transp. Syst..

[35] Zhang J., Che H., Chen F., Ma W., He Z. (2021). Short-Term Origin-Destination Demand Prediction in Urban Rail Transit Systems: A Channel-Wise Attentive Split-Convolutional Neural Network Method. Transp. Res. Part C.

[36] Ke J., Qin X., Yang H., Zheng Z., Zhu Z., Ye J. (2021). Predicting Origin-Destination Ride-Sourcing Demand with a Spatio-Temporal Encoder-Decoder Residual Multi-Graph Convolutional Network. Transp. Res. Part C.

[37] Huang Z., Wang D., Yin Y., Li X. (2022). A Spatiotemporal Bidirectional Attention-Based Ride-Hailing Demand Prediction Model: A Case Study in Beijing During COVID-19. IEEE Trans. Intell. Transp. Syst..

[38] Zhao J., Zhang R., Sun Q., Shi J., Zhuo F., Li Q. (2024). Adaptive Graph Convolutional Network-Based Short-Term Passenger Flow Prediction for Metro. IEEE Trans. Intell. Transp. Syst..

[39] Abbasimehr H., Shabani M., Yousefi M. (2020). An Optimized Model Using LSTM Network for Demand Forecasting. Comput. Ind. Eng..

[40] Yu B., Yin H., Zhu Z. Spatio-Temporal Graph Convolutional Networks: A Deep Learning Framework for Traffic Forecasting. Proceedings of the Twenty-Seventh International Joint Conference on Artificial Intelligence.

[41] Sattarzadeh A.R., Kutadinata R.J., Pathirana P.N., Huynh V.T. (2025). A Novel Hybrid Deep Learning Model with ARIMA Conv-LSTM Networks and Shuffle Attention Layer for Short-Term Traffic Flow Prediction. Transp. A Transp. Sci..

